# An Evaluation of Current Preventative Measures Used in Equine Practice to Maintain Distal Forelimb Functionality: A Mini Review

**DOI:** 10.3389/fvets.2021.758970

**Published:** 2021-11-02

**Authors:** Emily J. Clarke, Alex Gillen, Agnieszka Turlo, Mandy J. Peffers

**Affiliations:** ^1^Institute of Life Course and Medical Sciences, Musculoskeletal and Ageing Science, Liverpool, United Kingdom; ^2^Philip Leverhulme Equine Hospital, Institute of Veterinary Science, University of Liverpool, Liverpool, United Kingdom

**Keywords:** equine, joint, distal limb, preventative, osteoarthritis, tendon, function

## Abstract

Horses are used in a variety of equestrian disciplines predisposing them to musculoskeletal injury or disease including osteoarthritis and tendinopathy. As a result, a number of preventative measures are used within equine medicine and husbandry, ranging from therapeutic shoeing to the use of nutraceuticals. Despite their popularity and routine use evidence base and clinical outcomes are variable, bringing into question the efficacy of these prophylactic measures. In recent years a small number of studies have been performed examining the effect of specific strategies in order to quantify the preventative and protective claims such modalities have on joint and forelimb health. Few have robustly demonstrated a capacity to protect the limb by reducing inflammation, or promoting regenerative pathways. This review focusses on performance horses specifically, and the resounding theme that emerges in current research is the need for longitudinal studies to inform scientific conclusions surrounding single and multi-modal use. Furthermore, there is a requirement to prioritise evidence-based medicine to inform optimal clinical practice.

## Introduction

**Figure 1:**
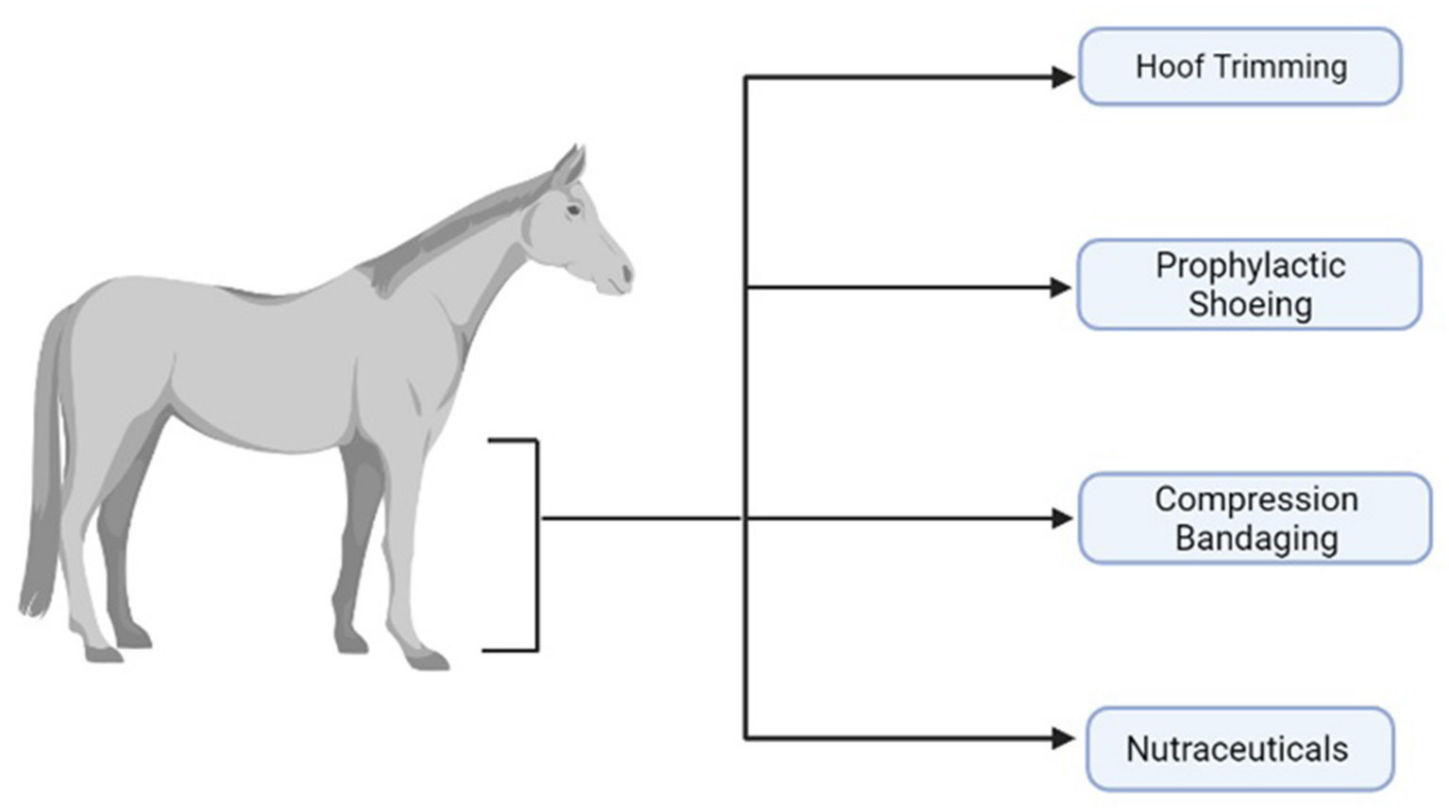
A visual summary of some of the prevention modalities used in an attempt to maintain equine distal forelimb functionality. Created with BioRender.com.

The equine musculoskeletal system has significant demands placed on it (1), specifically articular structures and supporting tissues, leaving it vulnerable to injury and disease development that results in pain, lameness, and reduced mobility. Consequently, numerous preventative modalities are being used in an attempt to maintain optimum athletic performance and potentially reduce the onset of musculoskeletal disease. The forelimb incorporates the carpus, a compound joint, composed of three articulations; the antebrachiocarpal joint; the middle carpal joint, and the carpometacarpal joint, commonly referred to as the carpal joint in a research setting (2). Soft tissue components include intercarpal ligaments, collateral ligaments, a fibrous joint capsule, and the palmar carpal ligament. The metacarpophalangeal joint is comprised of four bones; the third metacarpal bone, the proximal phalanx, and the paired proximal sesamoid bones (3). As the carpal and metacarpophalangeal regions are imperative for equine locomotion, they can be highly susceptible to injury and disease, most notably tendinopathies and osteoarthritis. Tendon injury is a common impairment that can compromise the stability of the joint capsule, which can result in excessive wear due to uneven force distribution. When the tendon heals, it becomes biomechanically inferior to the original structure, increasing the likelihood of re-injury. Osteoarthritis (OA) is a common disease of the joint, and is considered to be responsible for up to 60% of all lameness cases (4). Osteoarthritis is a progressive degenerative pathology, characterised by an imbalance in metabolic activity, resulting in articular degradation, osteophyte formation, synovitis and subchondral bone sclerosis (5, 6). Various preventative modalities are often used, including but not limited to foot trimming, therapeutic shoeing, compression bandaging, and nutraceuticals, in order to minimise damage to the associated structures (Figure 1). It should be highlighted that these preventative strategies are by no means exhaustive, and other modalities exist, such as physiotherapy, however this is well documented within literature. This “mini” review aims to evaluate the scientific evidence from the past 10 years surrounding preventative measures used in equine practice to maintain distal forelimb functionality. Both Web of Science and PubMed were used in order to identify appropriate literature, whereby search parameters included literature from the previous 10 years, following specific key terms, including “equine”, “musculoskeletal” and specific prevention modality, as shown in Figure 1.

## Hoof Trimming

Equine hoof trimming aims to maintain a healthy, functional hoof and prevent lameness, supporting articular structures and subsequent soft tissues within the limb (7). There are multiple hoof trimming strategies developed based on the dynamic-functional, natural-individual or static-geometric reference (8).

A study by Maeder et al. (9) described, using a pressure measurement system, that initial contact and breakover showed marked inter-individual variability amongst the 70 warmblood horses included in the study. Initial contact and breakover are often parameters measured during the stance phase as they are regarded as crucial references for optimised trimming practices, accounting for the high strain experienced by the distal limb. It was found that initial contact and centre of force were the most sensitive parameters affected by foot trimming, regardless of trimming practice, and that no significant long-term effects were concluded. The findings were thought to be determined by limb and body conformation, however, no significant long-term effects were concluded. In addition, Leśniak et al. (7) conducted a study that explored the linear and angular hoof variations pre- and post-farriery. Leśniak et al. (7) used 17 hoof and distal limb measurements from lateromedial and dorsopalmar digital photographs from 26 horses. They found that trimming practices lead to a reduction of the dorsal wall, and weight bearing resulted in increased vertical orientation of the hoof, improving dorsopalmar alignment. This highlights the potential of farriery to have substantial effects on the musculoskeletal system; correcting impaired conformation and reducing the excessive load on structures. Ultimately this resulted in the recommendation of regular 4–6-week farriery appointments to provide optimal protection to palmar limb structures, such as the deep digital flexor tendon and metacarpophalangeal joint. However, this recommendation was formed despite the lack of significant results, and large inter-individual variation.

Many factors may affect hoof growth and development, which may subsequently affect other areas of the distal limb, including the joints of the forelimb, due to altered and uneven weight distributions. These factors include: breed, diet, disease, height, pasture quality and lameness. Moeller et al. (10) examined the *in vitro* effect of hoof wall thinning on hoof structure and functionality, leading to increased deformability and resulting in damage to laminar tissue. This was undertaken using paired cadaver forelimbs of 12 horses. Each pair had the hoof wall from a single limb thinned by 25%. The contralateral hoof was used as a control within the study. Trimming the hoof was found to result in deformability of the entire hoof capsule, postulated to reduce damage to suspensory apparatus in the distal limb. In addition, there was significant disruption to suspensory apparatus of the distal phalanx in untrimmed hooves. This suggested a thinning of the hoof wall can decrease disruption to laminar tissue, supportive of hoof trimming as a prophylactic measure. However, it should be noted that this study was conducted *in vitro* and not *in vivo*. Therefore, the effect of the sole and frog were not been included. These structures may also be overloaded, and have a significant effect on the health and functionality of the distal limb and its joints (10). As such, the study does not reflect the physiology of live horses, therefore results may be affected by variables such as body and limb conformation, hoof shape, tension in tendons and ligaments, blood flow of structures, and speed of locomotion.

## Prophylactic Shoeing

Prophylactic shoeing, or farriery, is often used in the management or prevention of orthopaedic conditions, steeped in the practical application of anatomical biomechanics. It is suggested that such practises can displace concussion, alter the locomotive interactions of the hoof with the ground, distribute forces more evenly through the distal limb, and stabilise movement within the distal interphalangeal joint (11). Historically, this preventative modality has been used in practice, and recommended by farriers and clinicians through empirical evidence and tradition. However, very few controlled scientific studies have been conducted to rigorously ascertain the therapeutic efficacy of such strategy (12). Multiple shoeing methods have been conceived, such as z-bar shoes (open area of shoe is intended to reduce weight bearing on the palmar area of the hoof, reducing trauma and promoting healing) (13), egg bar shoes (increase the weight-bearing surface and create a larger area of posterior support for the foot and leg) (14), toe wedges and heel wedges (used on the rear palmar aspect of a collapsed heel region of a hoof wall) (8). These interventions are postulated to promote distal limb health, through supporting the joints of the lower limb and reducing unwanted biomechanical forces applied by the farrier if adverse conformational traits are identified.

Scientific studies conducted in relation to prophylactic shoeing are limited in number. Hagen et al. (8) examined the forelimbs of 30 horses when barefoot, equipped with toe wedges and heel wedges at various angles, in order to examine the effect of hoof angulation on the dorsal metacarpophalangeal joint, deep digital flexor tendon, and superficial digital flexor tendon. It was found that the application of a toe wedge or heel wedge significantly affected the dorsal metacarpophalangeal joint. Despite this it was noted that individual toe conformation and the cross-sectional area of flexor tendons were likely to influence any effect seen on hoof angulation and the dorsal aspect of the metacarpophalangeal joint (8). Thus, any significant effects evident as a result of remedial shoeing must be considered with respect to individual toe conformation. However, it has not been quantified in relation to pathological state, and the effect this may have on the distal joints. In addition, there was no evidence demonstrating the effects of long-term angulation. Stutz et al. (14) explored the effect of four shoeing methods (flat open shoe, rockered bar shoe, egg bar shoe, and unshod) on gait, measuring forelimb kinematic variables using an inertial measurement unit system. It was found that 75% of horses demonstrated a significant change in measured output in the presence of shoes. This was irrespective of shoe type, and independent of shoe geometry. It was also found that there were no differences between shoes when quantifying the non-podal kinematic variables investigated (such variables are important as shoeing has an effect on subsequent gait and biomechanics), notably in agreement with previously published work (14). Studies relating to prophylactic shoeing require further investigation, as it needs to be determined if therapeutic shoeing has a quantifiable benefit. Studies have previously found shod vs. unshod can have a significant effect on the limb, however, significant effects have not been shown with different types of shoe.

## Compression Bandaging

Compression bandages are used regularly within the veterinary profession, be it for the treatment of surgical or traumatic wounds, and for protection of the distal limb. Additionally they may reduce fluid accumulation associated with inflammation in distal limb oedema by minimising the effect of swelling on the range of motion and joint functionality. Compression bandaging relies on the concept of the venous muscle pump (15). Here hydrostatic pressure is created in the peripheral circulatory system as a result of the cardiovascular action of the heart. Various other structures of the circulatory system, such as the arteries and arterioles also contribute towards the pressure. These systems are subsequently affected by the contraction and relaxation of the musculature in the equine distal limb. Effectiveness of this technique is highly dependent on the interface pressure applied, physical activity, and elastic properties of the bandaging material (16).

Canada et al. (17) measured the effect of distal limb sub-bandage pressure over 96 h in eight horses. A distal limb compression bandage was used with the inclusion of cotton roll compressed with brown gauze and elastic layers. Alternatively, a polo wrap was also used in conjunction with a pillow pad. They found that the polo wrap's effectiveness in reducing fluid accumulation was dependent on anatomical location but not time. It was also shown that pressure distribution was not uniform, and it was demonstrated that the polo wraps maintained pressure for 24 h, whereas the distal limb compression bandage maintained high pressures for 96 h, without the need for reapplication. It was highlighted that, with regard to the application of compression bandages, there was variability in the technical skills the owners had. In an observational study by the same group on eight healthy adult horses used six bandaging techniques. These were the distal limb compression bandage, double layer bandage, inner sanctum bandage, carpal compression bandage, tarsal compression bandage, and adhesive elastic carpal bandage. During the study, sub-bandage pressures were measured using a picopress compression system. It was found that distal limb compression and inner sanctum bandaging resulted in significantly higher pressures compared with the distal limb compression bandage. Additionally, pressure was found to decrease significantly after walking with the use of a carpal compression bandage (18). Ultimately, the study concluded that clinician preference and reasoning should guide bandaging protocols for the equine limb, emphasising that the adequate pressure ranges required to reduce oedema are still to be determined. As a result, further work must quantify the most optimal pressure ranges to manage distal limb oedema, as well as the most appropriate duration of treatment, while also determining the clinical efficacy.

## Nutraceuticals

The nutraceutical industry is a growing area of preventive strategy in all companion animals, including the horse. Horse owners frequently supplement their horse's diets with various products in order to optimise equine health. A nutraceutical product can be defined as a substance which has a physiological benefit on the body, and that may provide prevention against chronic disease, such as the management of equine osteoarthritis (19). They often provide what is perceived as additional vitamins, minerals and electrolytes. However, perceptions surrounding their use and scientific evidence of their benefit are varied, with many companies claiming to enhance performance or alleviate health issues despite no significant evidence-based studies to support these claims. In addition, such modalities are not subject to robust regulations, and many are not assessed for safety or efficacy. Murray et al. (20) conducted a survey of owners and professionals associated with the Irish equine industry. The survey had 134 responses (70% non-professionals and 30% professionals). Most notably 98% of professionals included a supplement in their horse's diet, whereas 86% of non-professionals gave a supplement. Joint supplements were the most commonly used supplement by all survey participants. Additionally, 53% of respondents sought advice from a feed merchant, followed by their veterinarian (46%). Ninety-three percent of all respondents thought that nutraceuticals, such as feed supplements, had to meet legal standards, with 92% believing that supplements were always tested on horses before going to market.

It is often reported that the earliest recognisable feature of joint degeneration is loss of glycosaminoglycans from articular cartilage. As such, joint supplements are often used in an attempt to replace these with glycosaminoglycans thought to be chondroprotective (21). These include chondroitin sulphate, glucosamine and hyaluronan. Chondroitin sulphate has reportedly resulted in the inhibition of degradative enzymes, and an increase in anti-inflammatory activity (22). Glucosamine is an amino-monosaccharide and a precursor of the disaccharide units of articular cartilage glycosaminoglycans. Chondrocytes synthesise glucosamine from glucose but when glucosamine is available it is preferentially incorporated into cartilage (22). Hyaluronan is a key component of synovial tissue and synovial fluid, functionally serving as a lubricant and shock absorber (23). These are often the constituent components of supplements available for equines, and are muted to be incorporated into supplements due to their potential mechanisms of joint disease prevention.

Much et al. (24) evaluated the use of oral joint supplementation on gait kinematics and biomarkers of cartilage metabolism. Twenty horses were used in the study, stratified by age, sex, bodyweight and lameness scores. Horses were randomly assigned one of two dietary treatments, a 100 g placebo, or 100 g of joint supplement containing glucosamine, chondroitin sulphate, hyaluronic acid, methylsulfonylmethane, turmeric, resveratrol, collagen, silica, and boron. It was found that horses treated with the supplement had an increased range of motion in the hock at walk and trot compared with control groups. This was suggestive of the hock being sensitive to biomechanical changes due to supplementation. However, no change was observed between groups in serum and plasma biomarkers including a lack of alteration in the concentration of collagen metabolites, and no change in systemic markers of inflammation during the 28-day study period. Further to this, Gugliandolo et al. (25) investigated oral supplementation with ultra-micronized palmitoylethanolamide for joint disease and lameness management in four jumping horses. Four jumping horses were evaluated for non-responsive lameness, which resulted in them being withdrawn from show jumping competitions. Lameness was determined with the use of radiographic assessment, flexion tests, and lameness evaluation in accordance with the guidelines of American Association of Equine Practitioners. After 4 months of treatment it was determined that all horses showed remission of lameness, resulting in reintroduction to work and show jumping competitions, without disease recurrence. However, it should be noted that there was no appropriate control for this study and as such this makes the study evidence weak.

Van de Water et al. (26) measured the preventative effects of two nutraceuticals on experimentally induced synovitis in horses. Twenty-four standardbred horses were allocated a supplement, either: multi ingredient (28 days), collagen hydrolysate (60 days), meloxicam (4 days) or a placebo (60 days). Synovitis was induced by intra-articular injection of 0.5 ng of lipopolysaccharide into the right intercarpal joint. Subsequently blood and synovial fluid samples were analysed. It was found that both nutraceutical groups resulted in significantly lower synovial fluid total protein, total nucleated cell count and prostaglandin E2 in comparison with placebo. However, no statistical differences were observed between treatment groups for interleukin 6, matrix metalloproteinases and glycosaminoglycans. The study concluded that the use of nutraceuticals could reduce inflammation in a model of joint synovitis. However, the nutraceuticals used did not alter cartilage turnover, resulting in biomarkers associated with the development of joint diseases remaining elevated. Results of the studies described above leads to the question: should nutraceuticals be subject to stricter regulations, research and efficacy standards prior to being taken to market, in order to substantiate the claims made by their manufacturers?

## Conclusion

There are multiple preventative modalities used in equine veterinary medicine that aim to maintain the functionality of the forelimb. The lack of robust scientific studies is evident, with limited published literature within the last 10 years and often contradictory findings, despite the increasing popularity of the discussed modalities. Amongst the papers that have been published distinct themes emerge, such as the effect of individual variability and the number of confounding variables that may affect the efficacy of such modalities, such as: breed, age, conformation, sex, and owner compliance. The importance of robust experimental design is emphasised, including a longitudinal approach to quantify the prevention of disease or injury. This review highlights the importance of evidence-based medicine, and its place in driving forward veterinary practice standards. It has become apparent that in many cases joint injury and disease cannot be effectively prevented, substantiating the need for research into disease pathophysiology and potential therapeutics. This could result in improved clinical outcomes than those that currently exist, promoting recovery and enabling a complete return to work, fundamentally prioritising animal health and welfare.

## Author Contributions

EC, AG, AT, and MP worked to formulate review concept. EC conducted literature review and wrote manuscript. All authors revised the draft critically and read and approved the final submitted manuscript.

## Funding

EC is a self-funded PhD student. MP is funded through a Wellcome Trust Intermediate Clinical Fellowship (107471/Z/15/Z). AT is funded by Horserace Betting Levy Board Equine Post-Doctoral Fellowship (VET/2020−2 EPDF 8). Our work is also supported by the Medical Research Council (MRC) and Versus Arthritis as part of the MRC Versus Arthritis Centre for Integrated research into Musculoskeletal Ageing (CIMA).

## Conflict of Interest

The authors declare that the research was conducted in the absence of any commercial or financial relationships that could be construed as a potential conflict of interest.

## Publisher's Note

All claims expressed in this article are solely those of the authors and do not necessarily represent those of their affiliated organizations, or those of the publisher, the editors and the reviewers. Any product that may be evaluated in this article, or claim that may be made by its manufacturer, is not guaranteed or endorsed by the publisher.
